# Socioeconomic position, family context, and child cognitive development

**DOI:** 10.1007/s00431-024-05482-x

**Published:** 2024-03-14

**Authors:** Llúcia González, Maja Popovic, Marisa Rebagliato, Marisa Estarlich, Giovenale Moirano, Florencia Barreto-Zarza, Lorenzo Richiardi, Enrique Arranz, Loreto Santa-Marina, Daniela Zugna, Jesús Ibarluzea, Costanza Pizzi

**Affiliations:** 1 Spanish Consortium for Research On Epidemiology and Public Health (CIBERESP), Madrid, Spain; 2 JRU in Epidemiology, Environment and Health FISABIO-UJI-UV, Valencia, Spain; 3 Cancer Epidemiology Unit, Department of Medical Sciences, University of Turin and CPO Piemonte, Turin, Italy; 4 Predepartamental Unit of Medicine of Universitat Jaume I Castellón de La Plana, Castelló, Spain; 5 Nursing and Chiropody Faculty of Valencia University, Valencia, Spain; 6 Faculty of Psychology, Department of Basic Psychological Processes and their development, HAEZI Research Group, University of the Basque Country (UPV/EHU), San Sebastian, Spain; 7 Sub-Directorate for Public Health and Addictions of Gipuzkoa, Ministry of Health of the Basque Government, San Sebastian, Spain; 8 Environmental Epidemiology and Child Development Group, Biogipuzkoa Health Research Institute, San Sebastian, Spain

**Keywords:** Socioeconomic position, Cognitive development, Family context

## Abstract

Socioeconomic position (SEP) may have different effects on cognitive development and family context could play a role in this association. This work aimed to analyse the role of socioeconomic positions, measured via various indicators collected longitudinally, in cognitive development at 7–11 years of age, evaluating the role of family context as a potential mediator. The study sample included 394 and 382 children from the INMA Gipuzkoa and Valencia cohorts, respectively. SEP indicators were assessed during pregnancy (family social class, parental education, employment, and disposable income) and at 7 (Gipuzkoa) and 11 (Valencia) years of age (At Risk of Poverty or Social Exclusion (AROPE)). Family context and cognitive development were measured with the Haezi-Etxadi Family Assessment Scale 7–11 (HEFAS 7–11) and Raven’s Coloured Progressive Matrices (Raven’s CPM), respectively. Linear regression models were developed to assess the relationships between (a) SEP-family context, (b) SEP-cognitive development, and (c) family context-cognitive development, adjusting for a priori-selected confounders. Simple and multiple mediation analyses were performed to explore the role of family context in the SEP-cognitive development relationship. Lower SEP was related with a lower cognitive score, this association being particularly robust for family social class. SEP indicators were related to subscales of family context, in particular those regarding cognitive stimulation, parental stress, and parenting. A relationship was also found between these three subscales and child cognitive development, mediating the effect of family social class on child cognition by 5.2, 5.5, and 10.8%, respectively, and 12.0% jointly.

*    Conclusion*: Both family SEP and context contribute to a child’s cognitive development. Equalising policies and positive parenting programmes could contribute to improving cognitive development in children.

**Table:** 

**What is Known:** *• Parental social class, education, and employment status have been widely employed to measure socioeconomic position*.	
**What is New:** *• This work focuses on standard measurements of socioeconomic position but also other economic indicators such as the EHII and AROPE, and their effect on child cognitive development and family context*. *• Promotion of cognitive and linguistic development, parental stress and conflict, and parental profile fostering child development mediated the effect of family social class on cognitive development*.

**Supplementary Information:**

The online version contains supplementary material available at 10.1007/s00431-024-05482-x.

## Introduction

Cognitive development is the growth and maturation of thought processes [1]. One of the most employed approaches to understand cognitive development is those explored by Piaget [2], which defined cognitive development as a continuous process starting with the sensorimotor stage (birth to approximately 2 years of age) and ending with the formal operation stage (around 11 and 20 years of age). These periods of time are indicative, and one of the most interesting periods is the formal operations stage (7–11 years of age) when children are less egocentric and more focused on tasks and capable of solving complex problems [2, 3]. Socioeconomic position (SEP) amplifies differences in child cognitive development [4] and has been traditionally measured via social class, education level, or/and employment status [5].

The political agenda of the European Union (EU) has provided the At Risk of Poverty or Social Exclusion (AROPE) rate [6], a useful multidimensional tool to compare cohort samples consisting of the general population of European countries. However, the AROPE indicator includes self-reported family income, which could lead to biased responses [7]. Family income is not easy to measure, and the total income of a family should be considered only after tax deductions and social transfers standardised by household size and composition (total disposable income) [8]. Total disposable income varies among households, and its calculation could be useful to estimate family investment. The Equivalised Household Income Indicator (EHII) [9] overcame some of the difficulties deriving from total disposable income with data from the European Statistics on Income and Living Conditions (EU-SILC) [10] and the characteristics of the study participants.

In addition to the potential effects of SEP, events occurring within the family context could influence cognitive development [11]. The ecological systems theory [12] delves into family context and its effect on child development. This theory emphasises the interactive nature of child development that takes place based on regular relations with a consistent pattern and describes several nested layers around the child. The outer layer (exosystem) is where a society’s political, social, and economic characteristics are placed. Between the outer layer and the child, there are smaller systems (microsystems, such as family, friends, and school, among others) and the bonds between them (mesosystem), which jointly condition the effect of external layers [11, 12] on cognitive development.

Families with greater social vulnerability (those with less income, lower social class, or education) spend less time and resources on their children’s education, engage in fewer activities [13], and provide less and poorer cognitive and socioemotional scaffolding [14]. Scaffolding is defined as the intentional guidance and support offered by parents to their children in a specific task adjusting to their level of development [15]; a lack of sensitive scaffolding may result in a less stimulating context for cognitive development [16–19]. This family dynamic and the perception of the quality of the neighbourhood wherein the family is located [17, 20] are considered family investments [19]. Parents also establish practices to foster the child’s socioemotional scaffolding, which is conceptualised as the ability to apply the knowledge, attitudes, and skills necessary to understand and regulate emotions and set and achieve positive personal and academic goals [21]. It is crucial for critical brain development and emerging cognitive ability [22, 23].

Economic resources and cognitive and socioemotional scaffolding are transmitted through parenting (the attitudes and practices that parents deploy to care for their children) [20, 24, 25]. Parenting is influenced by perceptions and beliefs about parenting itself [24, 26] and the level of parental stress [27, 28]. Both factors seem to be involved in the family stress model, which shows that more stressed parents have greater difficulties in providing good-quality cognitive and socioemotional scaffolding [29] and, subsequently, promoting cognition [28].

In previous works, we explored SEP indicators (parental social class, education, and employment) and their relation to child cognitive development at 5 years of age [30], and also the effect of AROPE and family context on children’s mental health [7]. However, up to date, no study has compared classic SEP indicators (social class, parental education, and employment), the EU AROPE, and the EHII exploring their individual effect on child cognition, in a family context model. The aim of this work is novel and fourfold: (a) to assess the relationship of indicators reflecting different facets of SEP and cognitive development in formal operations stage (at 7 and 11 years of age) in the Gipuzkoa and Valencia cohorts, respectively; (b) to analyse the association between SEP indicators and family context; (c) to analyse the relationship between family context and cognitive development; and (d) to estimate the potential mediating role of family context in the relationship between SEP and cognitive development.

## Methods

### Study design and population

**Fig. 1:**
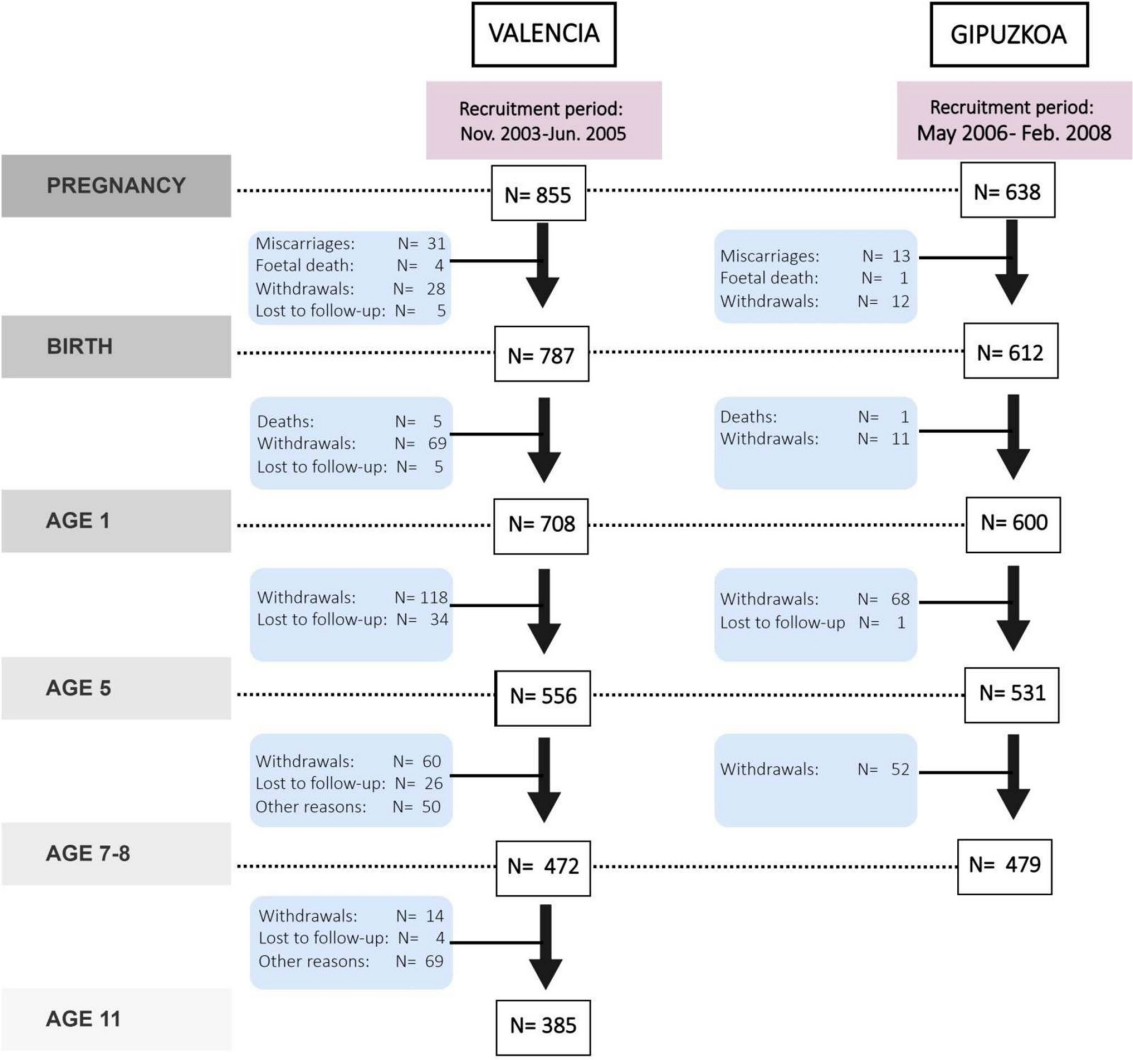
Numerosity and follow-up visits

The INMA study is a Spanish population-based mother-and-child multicentre cohort study set up in 2003 and composed of seven cohorts (Ribera d’Ebre, Granada, Menorca, Valencia, Sabadell, Asturias, and Gipuzkoa). Our study uses data from INMA Valencia and Gipuzkoa. Recruitment and subsequent procedures are described elsewhere [31]. Pregnant women were recruited during their first prenatal visit to their reference hospital before week 13 of gestation. The inclusion criteria were at least 16 years of age, 10–13 weeks of gestation, singleton pregnancy, intention of undergoing follow-up and delivery at their reference centre, and no communication impediment. In total, 855 women from Valencia (between November 2003 and June 2005) and 638 from Gipuzkoa (May 2006–February 2008) were recruited. Follow-up visits and sample evolution are described in Fig. 1. Cohorts were approved by local institutional ethical review boards, and participants gave their consent. This study conforms to the principles embodied in the Declaration of Helsinki.

### Exposure variables

#### Standard measurements of socioeconomic position

Education level, social class, and employment status were collected by interviewer at pregnancy for both parents who agreed to participate. The choice of these variables was based on the typical recommendations for measuring socioeconomic position in birth cohort studies [5]. The employment of these variables is shared by most epidemiological studies, which allows comparability with the present work. No single-parent family was declared during pregnancy.

Education level was defined as the highest level completed and classified with the International Standard Classification of Education 1997 (ISCED-97): up to primary (2C)/secondary (2A, 2B)/university (3 or higher). A combined variable for education of both parents was created based on if they were highly educated (having a university degree): (i) both parents, (ii) one, and (iii) none.

Social class was defined using an adaptation of the National Occupations Classification update (CNO-94) [32]. If a parent was unemployed when interviewed, the most frequent occupation of the last 10 years was considered. A combined variable for social class was elaborated, considering the highest social class of both parents, with the following categories: (i) highest (I + II), (ii) middle (III), and (iii) lowest (IV + V). For mediation analyses, to allow comparisons, the first and second categories were collapsed and compared to the lowest social class, which was equivalent to manual workers.

Employment status was defined as being employed, unemployed, or other situations (e.g. homemaker, student, retired). A combined variable for employment status of both parents was elaborated according to whether both were working or not.

#### The equivalised household income indicator (EHII)

The EHII is a socioeconomic indicator available for several birth cohort study members of the EU Child Cohort Network [33] and is a measure of total disposable household income—the sum of personal and household income of all the members of the household minus taxes—standardised for household size and composition. The EHII is an estimation of the standardised (for household size and composition) total disposable household income, obtained using EU-SILC data of 2011 and INMA data [9]. It was calculated for the pregnancy period and categorised according to the tertile of the 2011 Spanish EU-SILC [10] distribution of the equivalised total disposable income as (i) highest, (ii) middle, and (iii) lowest.

#### AROPE indicators

AROPE indicators were adopted by the European Union to monitor situations of extreme deprivation such as poverty and social exclusion. These indicators were employed in the present analysis to allow comparisons with classical indicators but also with EU-SILC statistics on AROPE [34] and to check if critical socioeconomic situations in middle childhood had an effect on cognitive development. They were assessed by structured questionnaires self-completed by parents in the 7- and 11-year follow-up visits of the Gipuzkoa and Valencia cohorts, respectively. Three dichotomous sub-indicators were calculated for each household [35], classifying them as at risk (vs. no risk) according to the following criteria:

1. Low work intensity (LWI): working < 20% of available hours of their working-age members the last year
2. Risk of poverty (RP): having < 60% of Spanish median income per consumption unit in the previous year
3. Risk of material deprivation (MD): lacking ≥ 3 necessary items from a list of 9 [30]

At Risk of Poverty or Social Exclusion (AROPE) [34] were those households fulfilling at least one of the three previous sub-indicators (LWI, RP, or MD).

### Outcome variable

A computerised version of Raven’s Coloured Progressive Matrices [36] was applied by a trained professional when children were aged 7 years in Gipuzkoa, and 11 in Valencia. It is a non-verbal assessment of fluid intelligence for people over 5 years of age and contains 36 items. Each item is an abstract pattern with a missing piece, the task consists of pinpointing the relationship between the elements of the system and solving the proposed problem [37]. It has strong construct validity and high discriminant validity [38]. In this analysis, we employed the number of items correctly answered by each participant (range 0–36).

### Potential mediators

At the follow-up visits of 7 (Gipuzkoa) and 11 years of age (Valencia), parents answered the Haezi-Etxadi Family Assessment Scale 7–11 (HEFAS 7–11) questionnaire, a self-reported measurement of family context and parenting skills. An exploratory and confirmatory factor analysis was performed including participants from both cohorts and five subscales were defined: promotion of cognitive and linguistic development (1. Cognitive), promotion of social and emotional development (2. Emotional), organisation of the physical environment and social context (3. Organisation), parental stress and conflict (4. Stress), and parental profile fostering child development (5. Parenting) (see Supporting Information Table 1 for further information). The subscales have good internal consistency, are independent between them, and assess a wide range of family context and parenting variables in 85 Likert-type items. Weighted scores were employed (ranges 16.76–100) for comparisons; higher scores imply higher quality of family context [39].

**Table 1:** Descriptive analysis of exposures, confounders, outcome, and potential mediators, stratified by cohort

			Total		Gipuzkoa		Valencia		
			*N*	%	*N*	%	*N*	%	*p*-value^a^
Exposures									
Family social class (pregnancy)	Higher (SC I + II)		310	39.95	194	49.24	116	30.37	< 0.001
	Middle (SC III)		195	25.13	84	21.32	111	29.06	
	Lower (SC IV + V)		271	34.92	116	29.44	155	40.58	
		*Missing*	0						
Parental educational level (pregnancy)	Both parents highly educated		135	17.56	80	20.57	55	14.47	< 0.001
	One parent highly educated		260	33.81	167	42.93	93	24.47	
	None highly educated		374	48.63	142	36.5	232	61.05	
		* Missing*	7						
Parental employment status (pregnancy)	Both parents employed		618	79.64	342	86.8	276	72.25	< 0.001
	Not both employed		158	20.36	52	13.20	106	27.75	
		* Missing*	0						
The Equivalised Household Income Indicator (pregnancy; tertiles)	Highest		283	37.40	182	48.90	99	26.00	< 0.001
	Middle		381	50.30	177	47.10	204	53.50	
	Lowest		93	12.30	15	4	78	20.50	
		* Missing*	19						
AROPE (7–11 years)	No risk		620	83.20	351	93.90	269	72.50	< 0.001
	Risk		125	16.80	23	6.10	102	27.50	
		* Missing*	31						
Risk of poverty (7–11 years)	No risk		646	86.60	361	96.30	285	76.80	< 0.001
	Risk		100	13.60	14	3.70	86	23.20	
		* Missing*	30						
Low work intensity (7–11 years)	No risk		733	94.70	383	97.46	350	91.86	< 0.001
	Risk		41	5.30	10	2.54	31	8.14	
		* Missing*	2						
Material deprivation (7–11 years)	No risk		737	94.97	385	97.72	352	92.15	< 0.001
	Risk		39	5.03	9	2.28	30	7.85	
		* Missing*	0						
			Mean	sd^b^	Mean	sd^b^	Mean	sd^b^	*p*-value^c^
Outcome									
Correct of total answers (Raven’s CPM)			29.19	4.82	26.68	0.24	31.86	0.16	0.289
		* Missing*	23						
POTENTIAL MEDIATORS: HEFAS 7–11									
Subscale 1: Promotion of Cognitive and Linguistic Development			70.16	12.84	66.99	11.24	73.41	13.56	< 0.001
		* Missing*	11						
Subscale 2: Promotion of Social and Emotional Development			83.42	8.76	79.42	7.90	87.58	7.60	< 0.001
		* Missing*	18						
Subscale 3: Organisation of the Physical Environment and Social Context			88.1	7.35	86.55	6.88	89.69	7.49	< 0.001
		* Missing*	11						
Subscale 4: Parental Stress and Conflict			77.84	10.17	77.27	9.51	78.49	10.85	0.108
		* Missing*	54						
Subscale 5: Parental Profile Fostering Child Development			80.18	9.25	79.29	8.87	81.1	9.55	0.007
		*Missing*	19						
			Total		Gipuzkoa		Valencia		
			*N*	%	*N*	%	*N*	%	*p*-value^a^
Confounders									
Maternal country of origin	Spain		745	96.01	384	97.46	361	94.50	0.035
	Other		31	3.99	10	2.54	21	5.50	
			0						
Paternal country of origin	Spain		728	93.81	388	98.48	340	89.01	< 0.001
	Other		48	6.19	6	1.52	42	10.99	
			0						
Maternal age	< 25		30	3.87	4	1.02	26	6.81	< 0.001
	25–29		244	31.44	121	30.71	123	32.20	
	30–34		367	47.29	201	51.02	166	43.46	
	35 +		135	17.40	68	17.26	67	17.54	
		* Missing*	1						
Paternal age	< 26		29	3.74	5	1.27	24	6.28	< 0.001
	26–30		201	25.94	86	21.88	115	30.10	
	31–35		329	42.45	176	44.78	153	40.05	
	36 +		216	27.87	126	32.06	90	23.56	
		* Missing*	0						
Parity	0		437	56.31	223	56.6	214	56.02	0.679
	1		294	37.89	151	38.32	143	37.43	
	2 or more		45	5.80	20	5.10	25	6.50	
		* Missing*	0						
History of maternal anxiety	Yes		99	8.50	8	2.00	58	15.20	< 0.001
	No		709	91.5	386	98.00	323	84.80	
		* Missing*	1						
History of maternal depression	Yes		42	5.40	4	1.00	38	10.00	< 0.001
	No		733	94.5	390	99.00	343	90.00	
		* Missing*	1						
Sex	Female		396	51.03	198	50.25	198	51.83	0.667
	Male		380	48.97	196	49.75	184	48.17	
		* Missing*	0						
			Mean	sd^b^	Mean	sd^b^	Mean	sd^b^	*p*-value
Maternal intelligence (WAIS)			10.05	3.05	9.97	2.72	10.12	3.31	0.523
		* Missing*	88						
Number of siblings	0		144	18.73	36	9.30	108	28.27	< 0.001
	1		505	65.67	279	72.09	226	59.16	
	2 or more		120	15.60	72	18.60	48	12.57	
		* Missing*	7						
Maternal cohabitation at 7–11 years	With father		666	86.27	364	92.86	302	79.47	< 0.001
	With another partner		58	7.51	15	3.83	43	11.32	
	With their parents		43	5.57	13	3.32	30	7.89	
	Alone with child		5	0.65	0	0	5	1.32	
		* Missing*	4						
			Total		Gipuzkoa		Valencia		
			Mean	sd^b^	Mean	sd^b^	Mean	sd^b^	*p*-value
Main care provider	Mother		407	56.45	172	49.14	235	63.34	< 0.001
	Mother and other		248	34.40	144	41.14	104	28.03	
	Others without mother		66	9.15	34	9.71	32	8.63	
		* Missing*	55						

^a^*p*-value from a chi-squared test for differences between cohorts

^b^Standard deviation

^c^*p*-value from ANOVA for differences between cohorts

### Confounders

A full set of a priori confounders was considered for fully adjusted models: maternal parity, maternal history of pre-pregnancy anxiety or depression (reported by the mother as having a previous diagnosis), parental country of origin, and age were reported in pregnancy and collected in a questionnaire by a trained interviewer. Maternal intelligence was measured with the similarities subtest of the Wechsler Adult Intelligence Scale (WAIS-III) when the child was 5 years old [40]. Other family variables were collected by questionnaire at different time points, including main care provider (child age: 2 years), number of siblings (4–5 years), and maternal cohabitation (7–11 years).

### Analysis

For descriptive analyses, frequencies and percentages were used for categorical variables, while means and standard deviations were used for continuous variables. The present analysis was planned in two parts: regression models and mediation analysis. Regression models explored the following three associations: (a) SEP-cognitive development, (b) SEP-family context, and (c) family context-cognitive development. Linear regression models were employed, considering that our response variable and mediating variables were continuous variables. An analysis of the residuals was carried out, and it was observed that the assumptions were met to apply a linear regression. To assess the SEP-cognitive development relationship, three models were developed: (i) minimally adjusted for age, sex, and cohort; (ii) fully adjusted for confounders collected in pregnancy and maternal intelligence; and (iii) sensitivity analysis performed only for AROPE indicators was additionally adjusted for main care provider, number of siblings, and maternal cohabitation. To assess the associations of SEP-family context and family context-cognitive development, models were adjusted for confounders measured during pregnancy and maternal intelligence.

**Fig. 2:**
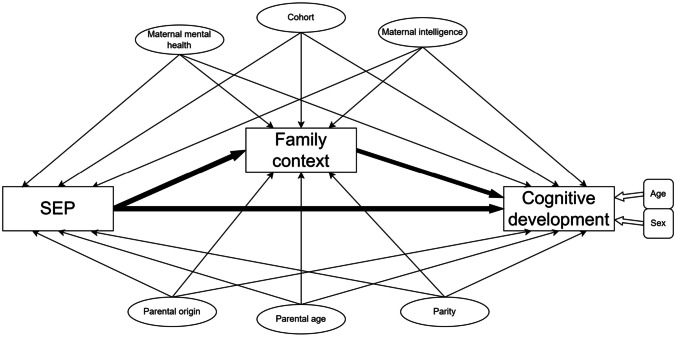
Directed acyclic graph for socioeconomic position, family context, and cognitive development

Mediation assessment was performed following the theoretical framework described by Bronfenbrenner [12] and further adapted to public health context by Pearce and collaborators [11]. According to this framework, SEP may have an effect on family context, and this environment may, in turn, have an effect on child development. Considering this, we employed a counterfactual mediation approach using the imputation method. Assumptions in our model are those standard of the counterfactual mediation approach [41, 42]. In Fig. 2 the directed acyclic diagram (DAG) describes the causal model, in which exposure (SEP), mediator (family context), and outcome (cognitive development) are drawn in squares, and direct and mediating pathways in bold arrows. Confounders are represented in circles, and their relation to the principal variables is pictured in plain arrows. Determinants of the outcome (sex and age) are placed in rounded squares and their relation to the outcome is specified in white arrows. Natural conditional effects were provided for single (using only one subscale at a time as a mediator for each model) and multiple mediator models (modelling multiple subscales at the same time). Mediation was further adjusted for the confounders of the fully adjusted models.

In all analyses, we explored the potential modification effects by sex and cohort; however, we did not find interactions by any of these variables. Missing data at baseline were treated using a complete case approach. To handle sample attrition, we used the inverse probability of participation weighting method. In particular, we fitted a logistic regression model with participation at follow-up as dependent variable and family social class, family education level, family employment status, family structure, equivalized household income indicator, child’s sex, maternal country of origin, parity, maternal age, smoking status at pregnancy, and maternal history of anxiety or depression as predictors of participation and derived the propensity stabilized weights. We then performed weighted regressions to derive the associational estimates of interest. Statistical analyses were performed with IBM SPSS Statistics (v.26), R and RStudio (v.4.1.3 and 2022.02.3 + 492, respectively), using the MASS, haven, foreign, ggplot2, and medflex packages. Figures 1 and 2 were designed with draw.io.

## Results

### Descriptive analysis

The study sample included 394 and 382 children from Gipuzkoa and Valencia, respectively. Regarding SEP indicators (Table 1), the family social class distribution was different across cohorts, with half of the Gipuzkoa sample being in the highest social class, while for Valencia, this category represented 30%. Regarding education, the most frequent situations were only one highly educated parent in Gipuzkoa (43%) and none in Valencia (61%). In most cases, both parents were employed. Families were mostly classified as being in the highest or middle tertile of the EHII, with few presenting AROPE. Considering all these socioeconomic characteristics, Valencia had a more deprived profile than Gipuzkoa.

Maternal intelligence was similar in both cohorts; most families were biparental, from parents born in Spain and mothers with no previous history of maternal depression (94.5%) or anxiety (91.5%). Most parents were 30–35 years old during pregnancy and, also, in most cases, participant children were the firstborns of two siblings. The most frequent main care provider was the mother, and 51% of children were female (Table 1). Considering cognitive development, cohorts presented similar mean values. Most of the HEFAS 7–11 scores differed between cohorts, with the Valencia cohort presenting higher mean scores (richer family context) (Table 1).

### SEP and cognitive development

**Fig. 3:**
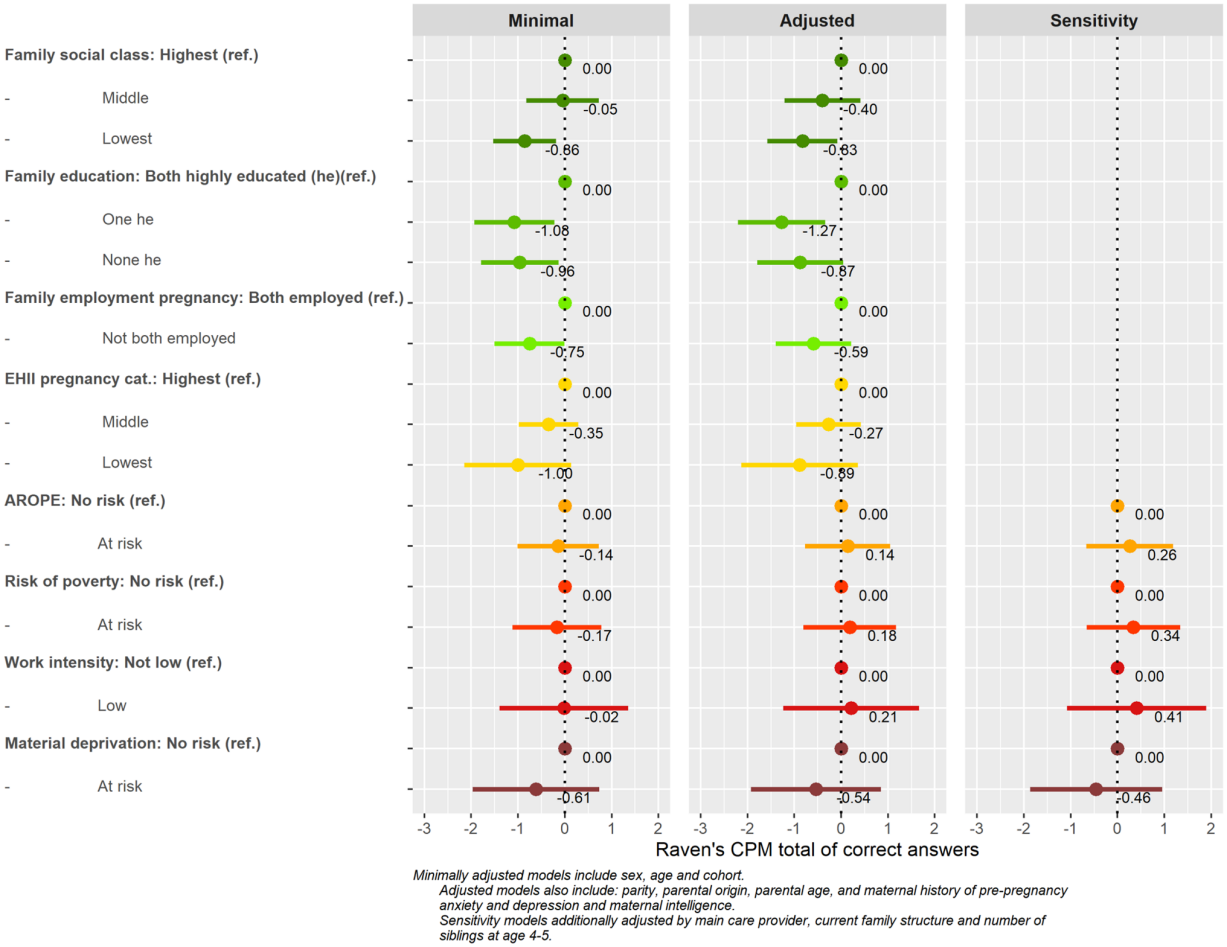
Minimal, adjusted, and sensitivity models

The relationships between SEP indicators and Raven’s CPM are represented in Fig. 3 and Supporting Table 2. Family social class (B[95%CI]) (middle vs. highest: − 0.05 [− 0.82, 0.73]), (lowest vs. highest: − 0.86 [− 1.53, − 0.18]), family education (one vs. both highly educated: − 1.08 [− 1.94, 0.22]), (none vs. both highly educated: − 0.96 [− 1.79, − 0.13]); and EHII (middle vs. highest: − 0.35 [− 0.98, 0.29]), (lowest vs. highest: − 1.00 [− 2.14, 0.14]) presented a social gradient in the minimally adjusted models, showing lower cognitive scores in more socioeconomically deprived families. Having at least one parent unemployed was also linked to lower cognitive scores. AROPE, risk of poverty or material deprivation showed a similar negative association although with 95%CI crossing the null value. The adjustment for confounders reduced the magnitude of the associations; however, the direction did not change for most SEP exposures, while for AROPE and risk of poverty were close to 0. In the adjusted models, the association of material deprivation remained similar to the minimally adjusted models. Sensitivity analyses did not greatly change the results for AROPE indicators when compared to the fully adjusted models. No modification effect of sex or cohort was found.

**Table 2:** Natural conditional effects for mediation models

	**Coefficient**	**95% CI**	
**Mediation through subscale 1: promotion of cognitive and linguistic development**			
Direct effect	− 0.62	− 0.96	0.71
Indirect effect	− 0.06	− 0.16	0.03
Total effect	− 0.68	− 1.03	0.64
**Mediation through subscale 4: parental stress and conflict**			
Direct effect	− 0.66	− 0.99	0.69
Indirect effect	− 0.08	− 0.19	0.08
Total effect	− 0.73	− 1.03	0.62
**Mediation through subscale 5: parental profile fostering child development**			
Direct effect	− 0.59	− 0.96	0.75
Indirect effect	− 0.14	− 0.29	− 0.03
Total effect	− 0.73	− 1.11	0.58
**Joint mediation through subscales 1, 4, and 5**			
Direct effect	− 0.56	− 0.85	0.85
Indirect effect	− 0.18	− 0.40	− 0.03
Total effect	− 0.74	− 1.04	0.61

### SEP and family context

**Fig. 4:**
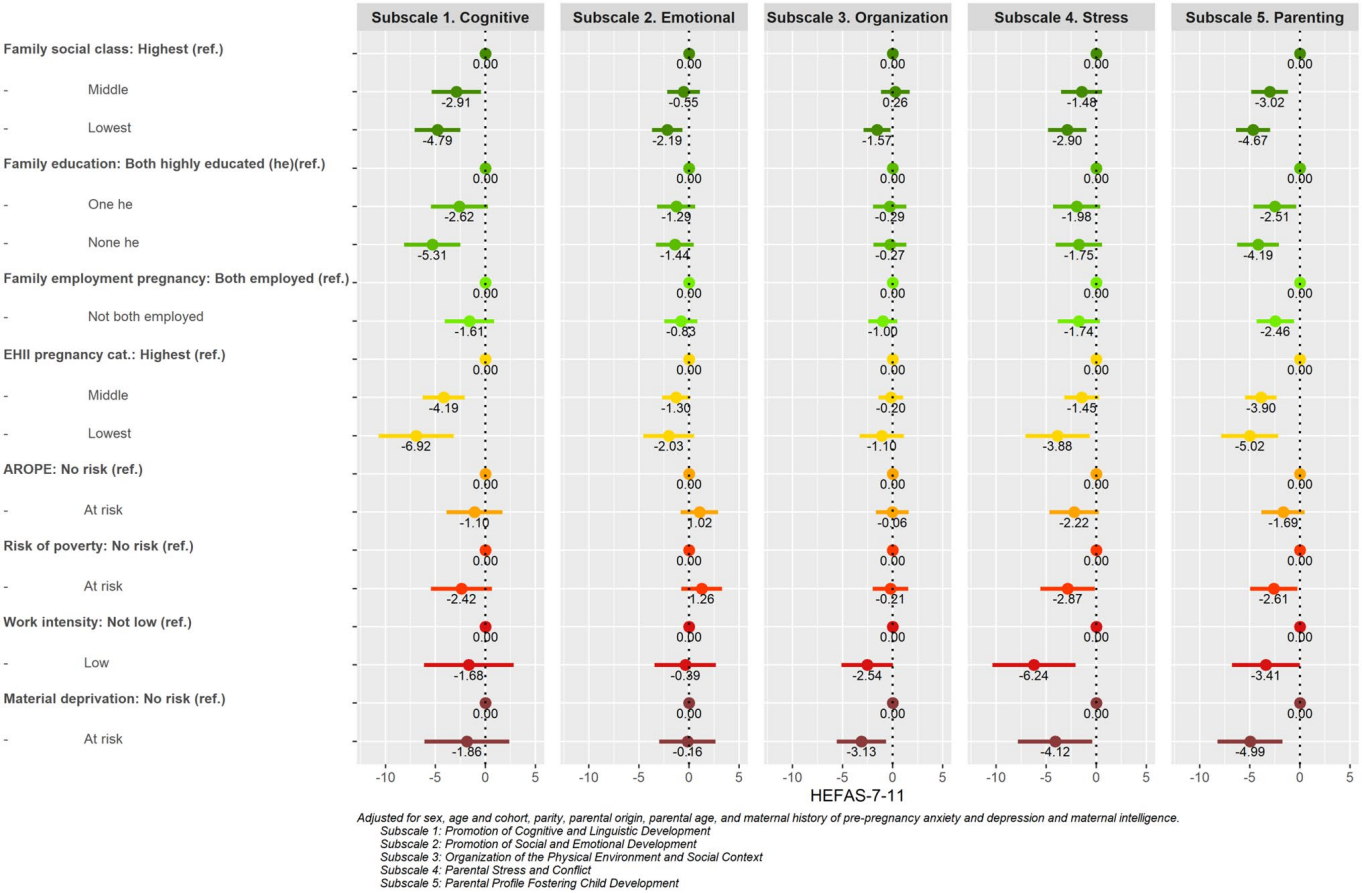
Sep relation to HEFAS 7–11

Figure 4 and Supporting Table 3 show the relationship between SEP indicators and family context. Subscale 1. Cognitive presents similar trends to those observed for cognitive scores, being inversely related to most SEP indicators, including family social class (middle vs. highest: − 2.91 [− 5.37, − 0.45]), (lowest vs. highest: − 4.79 [− 7.07, − 2.51]). There were no associations with SEP in the case of Subscales 2. Emotional and 3. Organisation. For Subscale 4. Stress, all SEP indicators show that more deprived families reported lower family context scores. This was particularly clear for EHII (middle vs. highest: − 1.45 [− 3.19, 0.28]) (lowest vs. highest: − 3.88 [− 7.10, − 0.66]), AROPE (− 2.22 [− 4.68, 0.25]), risk of poverty (− 2.87 [− 5.60, − 0.14]), low work intensity (− 6.24 [− 10.39, − 2.09]), and material deprivation (− 4.12[− 7.82, − 0.41]). The negative associations of all SEP indicators were particularly strong with Subscale 5. Parenting.

### Family context and cognitive development

**Fig. 5:**
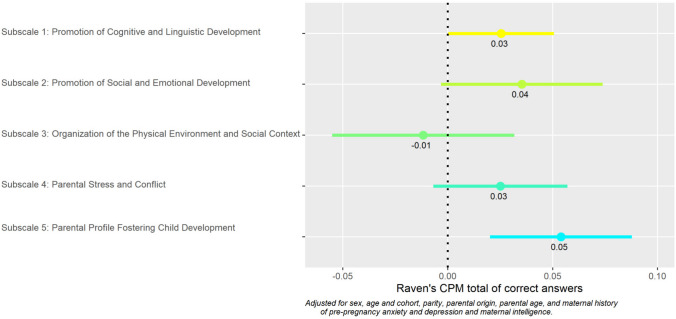
HEFAS 7–11 subscales and Raven’s CPM

Figure 5 shows the relation between HEFAS 7–11 and Raven’s CPM. Subscales. 1. Cognitive (0.03 [0.00, 0.05]), 4. Stress (0.03 [− 0.01, 0.06]), and 5. Parenting (0.05 [0.02, 0.09]) were more strongly associated with Raven’s CPM, and Subscales 2. Emotional and 3. Organisation did not show an association with child cognition.

### Mediation analysis

For mediation analysis, we selected the SEP indicator presenting the strongest association with cognitive development (family social class), and as mediators, the family context subscales associated to family social class and cognitive development (see Figs. 3 and 4 for more detail: Subscales 1. Cognitive, 4. Stress, and 5. Parenting). Natural conditional total, direct, and indirect effects are described for single and multiple mediations in Table 2. In the single mediation analysis, Subscale 1. Cognitive, presented the following estimates: direct effect was (B [95%CI]) (− 0.62 [− 0.96, 0.71]), and indirect effect was (− 0.06 [− 0.16, 0.03]). This subscale alone mediated up to 9.2% of the relationship between social class and Raven’s CPM. The single mediation for Subscale 4. Stress presented similar estimates (indirect effect (− 0.08 [− 0.19, 0.08])), mediating 5.8% of the SEP-child cognition effect. The percentage total effect of family social class on child cognition mediated by Subscale 5. Parenting was 19.8% (indirect effect (− 0.14 [− 0.29, -0.03])). The indirect joint effect with all three Subscales considered was (− 0.18 [− 0.40, − 0.03]), mediating 24.3% of the total effect. In all analyses, we explored the potential modification effects by sex and cohort, however, we did not find interactions by any of these variables.

## Discussion

This study explored the role of diverse SEP indicators in child cognitive development, considering standard measurements (such as social class, education, and employment), AROPE, and EHII. We further examined the role of family interactions as potential mediators between SEP and child cognitive development. Overall, we observed an inverse association between socioeconomic deprivation and cognitive development. The most consistent associations were observed for family social class. In addition, our findings suggest that in the relation between SEP and cognitive development, family context might play a relevant role. This mediation was especially strong in the case of parenting, and we found that cognitive scaffolding, parental stress, and parenting could jointly mediate 24.3% of this relationship.

We first aimed to assess the relationship between SEP indicators and cognitive development. Children from more economically deprived backgrounds scored lower in cognitive development. This was particularly evident for family social class, parental education, and employment, as well as for EHII, while for AROPE, the lower cognitive development scores presented a 95%CI crossing the null value. A Chinese work also separately explored different ways of measuring SEP, finding that they were directly related to cognitive development. However, among all SEP indicators examined, only education and occupation status (and not income) remained associated with child cognitive development when a family investment mediation path was tested [20]. Another very recent work from ABCD study analysing interactions between the environment, brain, and cognition and behaviour in children tested the effect of several predictors (more than 40 comprising several areas) and found that family income and caregiver education presented the greatest influence on brain’s functional network connectivity. This was especially important in hippocampus and thalamus, which are involved in long-term memory and perception-cognition processes, respectively, and therefore, the consolidation of cognitive development [43]. A recent study with US sample, presented a strong positive association of SEP and cognition, and almost a null effect of social mobility on cognitive development [44]. The most feasible explanation for this is known as *the latency model*, this is that the exposure to low SEP in critical windows of development could impair cognitive functioning [45].

Different socioeconomic indicators present facets of overall socioeconomic well-being and thus could have diverse effects on cognitive development. For example, employment status affects income but also time, lifestyle, and engagement in unhealthy habits [5]. Occupational social class may define the time schedule [32], risk exposures, ease of reconciling family and work life, and social prestige [46] and provide a chance of ranking individuals according to their income [32]. Total disposable household income, a direct measure of material resources, was estimated via the EHII, which showed associations similar to those of family social class, both with cognitive development and family context. AROPE allows us to compare participants with the general population periodically monitored by this index [34]. In the present work, we adjusted our analyses for a priori confounders associated with AROPE in a previous work [35], and which could be overshadowing the effect of AROPE. It is noteworthy that AROPE and child cognitive development were measured cross-sectionally and, as noted by some previous works, SEP is less associated with cognitive development [19, 20] if measured during childhood instead of pregnancy.

Furthermore, parental education, a measure of intellectual resources, was shown to be positively related to cognitive and language development [47]. It is not easy to disentangle the independent effect of each component of socioeconomic well-being as they tend to be highly correlated, providing slightly different facets of the same concept. We were also not able to clearly discern between the role of material and intellectual resources; however, given the consistent direction and magnitude of estimates for different SEP indicators, our results suggest that both types of resources are considered proxies of family investment and seem to play a role in child cognitive development [48].

Our second objective was to analyse the relationship between SEP indicators and family context. We observed that all SEP indicators considered were strongly associated with some family context subscales, finding that poorer SEP related to a less positive family context (less stimulating cognitive and linguistic context, higher stress and conflict, and less parental promotion of child development). A recent publication also found similar results, with better cognitive development as socioeconomic status increased [49]. Previous works observed that more highly educated parents provide contingent scaffolding [14]. A work examining the association of several SEP indicators with attitudes towards parental scaffolding (provision of materials, organised activities, helping with homework) also found a positive association [20]. Parental stress has also been previously linked to scarcity of resources [28]; one example is a research finding that past, but not current material hardship, increased stress, decreased couple relationship quality, and could provoke further generalised anxiety disorders [50]. Parents with a better SEP are considered to be more aware and involved in their children’s development [26]. A very recent study considering potential factors related to SEP and cognitive development observed that parents with higher education valued the efforts of their children more and believed in the importance of education [51]; this trend was also suggested by our analyses.

Our third aim was to evaluate the relationship between family context and cognitive development, and our fourth objective was to estimate the potential mediating role of family context in the association between SEP and cognitive development. In our work, family context played an important role in the relationship between social class and cognitive development. Cognitive and linguistic development, parental stress and conflict, and parental profile fostering child development appeared to be related to socioeconomic factors. These aspects of family interactions mediated around one fourth of the total effect of SEP on child cognitive development. In general terms, both family social class and family context were related separately to cognitive development. We also found that family social class was related to family context. We also found that parenting profile fostering child development, and the total joint mediation model, played an important role in the SEP-cognitive development association.

Other works assessing the effect of factors related to family context. In this way, a work with Italian sample discovered that home environment mediated the effect of SEP in cognitive development. Similarly, a recent publication found that family context and child’s assistance to nursery fully explained the effect of socioeconomic status on cognitive development [49]. Some works found some characteristics of family context, such as parental cognitive scaffolding (reading together, helping with homework, explaining the meaning of words, among others)[52] to mediate the effect of SEP on cognition. A systematic review indicated that scaffolding mediated the effect between poverty and cognitive development [53], and a study focused on income-to-needs ratios found that cognitive scaffolding mediated some of the effects of two SEP measures on cognitive functions [54]. A work from the Millennium cohort found that activities such as reading to children mediated the effect of economic deprivation on cognitive development [55]. Finally, another work, found that economic status, parental education, and employment were all positively associated with the provision of cognitively stimulating materials at home, and this factor was in turn related to cognitive ability [20]. Most of these works were carried out with younger samples than ours, and perhaps, provision of this stimulation could be more relevant for cognitive development at early rather than at middle childhood. In addition, this could also explain that this association was not strong in our analyses.

The family stress model seems to be related to child development [20, 28, 53]. Two previous studies found that stress did not mediate the effect of SEP on cognitive development [16, 47], however, one study reported mediation when considering linguistic development as the outcome [47]. An Australian work found that stress influences cognitive outcomes but that it might be less relevant than for non-cognitive outcomes. In the association between SEP and cognitive development, stress mediated 5–10% of the effect [52]. In our case, this association was not as strong as we expected, but it still mediated a similar share of the effect (5.8%) in comparison to the Australian publication.

Parenting profile fostering child development is the knowledge, beliefs, attitudes, and practices regarding child development and parenting. A systematic review of mediators of the relationship between poverty and cognitive development argued that the effects of poverty on cognitive development could be mitigated through parenting [53]. The mother–child relationship was found to mediate the effect of economic deprivation on child cognitive development in the Millennium cohort [55].

Similarly to our multiple mediation analysis, a German study analysed the effect of income and net worth on cognitive development, jointly considering several potential mediating factors: neighbourhood quality, educational norms and aspirations, mother–child interaction quality, family investment, and parental stress. They found that family investment, measured as the provision of materials and activities, was the most relevant factor in the mediation path [16], while in our analyses, the most important mediating factor was parenting profile fostering child development.

The present work has several limitations. Firstly, all cohort studies, especially those with long follow-ups, present sample attrition. In fact, our analysis sample was 58% of the initial sample and differed from the baseline sample (Spanish parents with a higher education level and social class were more frequent in the analysis sample). For this reason, we employed inverse probability weighting to adjust by potential sample attrition. Secondly, some of the exposure-outcome associations were estimated from cross-sectional data. In the mediation analysis, we hypothesised that family interactions and context are likely to precede cognitive functioning; however, the temporal relationship between the two cannot be established with certainty.

Our study also possesses several strengths: we considered a full range of variables from diverse follow-ups, collected mostly in a prospective manner. We considered different SEP indicators, which could reflect various socioeconomic dimensions and their effects on cognitive development and family context. We used HEFAS 7–11, which provides a full landscape of family context including contextual and interactive variables that are rarely available with such detail. In addition, we used an unbiased measurement of cognitive development based on a traditional and strong conception of cognition, which considers non-verbal visual stimuli to estimate a child’s deduction ability. Finally, this work employed data from children aged between 7 and 11 years, covering middle childhood, and from areas of diverse economic levels, providing additional robustness to our work.

Two groups of possible mechanisms of action and recommendations are feasible in this context. The first group should focus on direct mechanisms that education and economic policymakers should take into consideration: we have observed the impact of low SEP in cognitive development; for this reason, we recommend policies to improve SEP indicators in the population, for example, promoting free public and quality education, providing a minimum income to live with dignity, and improving public employment services, among others. We also have observed that better parenting skills and stimulation were related with higher cognitive performance. For this reason, the second group of actions, which are indirect mechanisms, should be focused on positive parenting. Practitioners should jointly work with teachers and educational psychologists to provide a safe space to ask doubts about development and upbringing. This could be realized with periodic open forums that locally respond to parent’s petitions, but one the most common tools employed are the positive parenting programmes, which supply parents with knowledge about child development, help them to manage stress, and encourage them to engage in high-quality parent–child interactions.

In conclusion, we found that different SEP indicators suggest that more socially deprived families have children with lower cognitive development, and in the case of family social class, this association had an important impact on both family context and cognitive development. These findings could be useful for both implementing equalising policies and fostering positive parenting. Future analyses could deepen in the explored relationship employing second-order factors from family context to allow interpretation of parenting processes.

### Supplementary Information

Below is the link to the electronic supplementary material.

Supplementary file1 (DOCX 51 KB)

## Data Availability

No datasets were generated or analysed during the current study.

## Abbreviations

AROPE: At Risk of Poverty or Social Exclusion
EHII: Equivalised Household Income Indicator
HEFAS 7-11: Haezi-Etxadi Family Assessment Scale
Raven’s CPM: Raven’s Coloured Progressive Matrices

## References

[1] American Psychological Association (APA) (2021) Cognitive development – APA Dictionary of Psychology. https://dictionary.apa.org/cognitive-development. Accessed 11 Nov 2021

[2] Babakr ZH, Mohamedamin P, Kakamad K (2019). Piaget’s cognitive developmental theory: critical review. Educ Q Rev.

[3] Berk LE (1998). Desarrollo del niño y del adolescente, 1^a^.

[4] Burneo-Garcés C, Cruz-Quintana F, Pérez-García M (2019). Interaction between socioeconomic status and cognitive development in children aged 7, 9, and 11 years: a cross-sectional study. Dev Neuropsychol.

[5] Dahlgren G, Whitehead M (2010) Estrategias europeas para la lucha contra las desigualdades sociales en salud: Desarrollando el máximo potencial de salud para toda la población - Parte 2 (publicado originalmente como Concepts and principles for tackling social inequities in health: Levelling up Part 2). Ministerio de Sanidad y Política Social (originalmente publicado por la Oficina Regional de la OMS para Europa)

[6] Eurostat (2021) Statistics | Eurostat. In: People Risk Poverty Soc. Exclusion Age Sex - New Defin. https://ec.europa.eu/eurostat/databrowser/view/ILC_PEPS01N__custom_1171301/default/table?lang=en. Accessed 26 Jul 2021

[7] González L, Estarlich M, Murcia M (2021). Poverty, social exclusion, and mental health: the role of the family context in children aged 7–11 years INMA mother-and-child cohort study. Eur Child Adolesc Psychiatry.

[8] Eurostat (2021) Glossary:equivalised disposable income. https://ec.europa.eu/eurostat/statistics-explained/index.php?title=Glossary:Equivalised_disposable_income. Accessed 22 Jun 2022

[9] Pizzi C, Richiardi M, Charles M-A (2020). Measuring child socio-economic position in birth cohort research: the development of a novel standardized household income indicator. Int J Environ Res Public Health.

[10] Eurostat (2023) Methodology - income and living conditions - Eurostat. https://ec.europa.eu/eurostat/web/income-and-living-conditions/methodology. Accessed 20 Mar 2023

[11] Pearce A, Dundas R, Whitehead M, Taylor-Robinson D (2019). Pathways to inequalities in child health. Arch Dis Child.

[12] Bronfenbrenner U (1994) Ecological models of human development. In: International Encyclopedia of Education, 2nd ed. Oxford: Elsevier, Freeman, NY

[13] Schaub M (2015). Is there a home advantage in school readiness for young children? Trends in parent engagement in cognitive activities with young children, 1991–2001. J Early Child Res.

[14] Mermelshtine R (2017). Parent–child learning interactions: a review of the literature on scaffolding. Br J Educ Psychol.

[15] Neale D, Whitebread D (2019). Maternal scaffolding during play with 12- to 24-month-old infants: stability over time and relations with emerging effortful control. Metacognition Learn.

[16] Dräger J, Pforr K (2022). The multiple mediators of early differences in academic abilities by parental financial resources in Germany. Adv Life Course Res.

[17] Li Y, Yang H, Luo L (2021). Poverty exposure and cognitive abilities of children in rural China: causation and the roles of family investments. Child Youth Serv Rev.

[18] de Neubourg E, Borghans L, Coppens K, Jansen M (2018). Explaining children’s life outcomes: parental socioeconomic status, intelligence and neurocognitive factors in a dynamic life cycle model. Child Indic Res.

[19] Simons LG, Wickrama K, a. S, Lee TK,  (2016). Testing family stress and family investment explanations for conduct problems among African American adolescents. J Marriage Fam.

[20] Zhang Y (2021). The role of socioeconomic status and parental investment in adolescent outcomes. Child Youth Serv Rev.

[21] Chance EK (2018) The effects of the ready for success classroom guidance program on the social-emotional skills and competence, reading proficiency, and promotion rate of third-grade students. Ph. D

[22] Polichetti P (2014) Fostering social-emotional development in K-3 classrooms

[23] Thielking M, Terjesen MD (2017). Handbook of australian school psychology.

[24] Roubinov DS, Boyce WT (2017). Parenting and SES: relative values or enduring principles?. Curr Opin Psychol.

[25] Bi X, Yang Y, Li H (2018). Parenting styles and parent–adolescent relationships: the mediating roles of behavioral autonomy and parental authority. Front Psychol.

[26] National Academies of Sciences E, Education D of B and SS and, Board on Children Y et al (2016) Parenting knowledge, attitudes, and practices. In: Parenting matters: supporting parents of children ages 0–8. National Academies Press (US) 27997088

[27] Blair C, Raver CC (2016). Poverty, stress, and brain development: new directions for prevention and intervention. Acad Pediatr.

[28] Masarik AS, Conger RD (2017). Stress and child development: a review of the Family Stress Model. Curr Opin Psychol.

[29] Barreto-Zarza F, Arranz-Freijo EB (2022). Family context, parenting and child development: an epigenetic approach. Soc Sci.

[30] González L, Cortés-Sancho R, Murcia M (2020). The role of parental social class, education and unemployment on child cognitive development. Gac Sanit.

[31] Guxens M, Ballester F, Espada M (2012). Cohort profile: the INMA–INfancia y Medio Ambiente–(Environment and Childhood) Project. Int J Epidemiol.

[32] Domingo-Salvany A, Regidor E, Alonso J, Alvarez-Dardet C (2000) Proposal for a social class measure. Working Group of the Spanish Society of Epidemiology and the Spanish Society of Family and Community Medicine. Atencion Primaria Soc Esp Med Fam Comunitaria 25:350 10905823

[33] EU Child Cohort Network. In: LifeCycle. https://lifecycle-project.eu/for-scientists/the-eu-child-cohort-network/. Accessed 29 Dec 2023

[34] Eurostat (2021) Glossary:At Risk of Poverty or Social Exclusion (AROPE). https://ec.europa.eu/eurostat/statistics-explained/index.php?title=Glossary:At_risk_of_poverty_or_social_exclusion_(AROPE). Accessed 13 Sep 2022

[35] González L, Estarlich M, Murcia M (2020). Risk of child poverty and social exclusion in two Spanish regions: social and family determinants. Gac Sanit.

[36] (2022) Vienna Test System - SCHUHFRIED. https://www.schuhfried.com/es/vienna-test-system/. Accessed 30 Dec 2022

[37] Raven J, Raven J (1998) Raven’s Coloured Progressive Matrices Manual. Harcourt Assessment, San Antonio, Texas, USA

[38] Antoniou F, Alkhadim G, Mouzaki A, Simos P (2022). A psychometric analysis of Raven’s Colored Progressive Matrices: evaluating guessing and carelessness using the 4PL item response theory model. J Intell.

[39] Barreto-Zarza F, Sánchez de Miguel M, Ibarluzea J (2021). Family context assessment in middle childhood: a tool supporting social, educational, and public health interventions. Int J Environ Res Public Health.

[40] Corral S (1999). WAIS-III.

[41] Vansteelandt S, Bekaert M, Lange T (2012). Imputation strategies for the estimation of natural direct and indirect effects. Epidemiol Methods.

[42] Zugna D, Popovic M, Fasanelli F (2022). Applied causal inference methods for sequential mediators. BMC Med Res Methodol.

[43] Zhi D, Jiang R, Pearlson G (2023). Triple interactions between the environment, brain, and behavior in children: An ABCD study. Biol Psychiatry.

[44] Johnson SB, Raghunathan RS, Li M (2022). Moving up but not getting ahead: family socioeconomic position in pregnancy, social mobility, and child cognitive development in the first seven years of life. SSM - Popul Health.

[45] Skoblow HF, Proulx CM, Palermo F (2024) Childhood socioeconomic position and later-life cognitive functioning in the U.S.: a critical review. Dev Rev 71:101104. 10.1016/j.dr.2023.101104

[46] Eurostat (2023) Reconciliation of work and family life - statistics. https://ec.europa.eu/eurostat/statistics-explained/index.php?title=Reconciliation_of_work_and_family_life_-_statistics. Accessed 24 Feb 2023

[47] Telias A, Narea M, Abufhele A (2022). A mediation analysis to disentangle relations between maternal education and early child development. Int J Behav Dev.

[48] Sohr-Preston SL, Scaramella LV, Martin MJ (2013). Parental Socioeconomic status, communication, and children’s vocabulary development: a third-generation test of the family investment model. Child Dev.

[49] Aranbarri A, Aizpitarte A, Arranz-Freijo E (2023). What influences early cognitive development? Family context as a key mediator. J Appl Dev Psychol.

[50] Daundasekara SS, Schuler BR, Beauchamp JES, Hernandez DC (2021). The mediating effect of parenting stress and couple relationship quality on the association between material hardship trajectories and maternal mental health status. J Affect Disord.

[51] Xu H, Zhang Z, Zhao Z (2023). Parental socioeconomic status and children’s cognitive ability in China. J Asian Econ.

[52] Khanam R, Nghiem S (2016). Family income and child cognitive and noncognitive development in Australia: Does Money Matter?. Demography.

[53] Saitadze I, Lalayants M (2021). Mechanisms that mitigate the effects of child poverty and improve children’s cognitive and social–emotional development: a systematic review. Child Fam Soc Work.

[54] Rosen ML, Hagen MP, Lurie LA (2020). Cognitive stimulation as a mechanism linking socioeconomic status with executive function: a longitudinal investigation. Child Dev.

[55] Kiernan KE, Huerta MC (2008). Economic deprivation, maternal depression, parenting and children’s cognitive and emotional development in early childhood. Br J Sociol.

