# Supplementary material for: Socioeconomic position, family context, and child cognitive development
Source: Eur J Pediatr. 2024 Mar 14;183(6):2571–85. doi: 10.1007/s00431-024-05482-x (PMC11098862; doi:10.1007/s00431-024-05482-x)
Supplement: Supplementary file 1 — Supplementary file1 (DOCX 51 KB) [file 431_2024_5482_MOESM1_ESM.docx]

# Supplementary material

## Supplementary methods 1: the Equivalised Household Income Indicator (EHII)

The EHII is based on external data from the 2011 European Surveys on Income and Living Conditions (EUSILC) and internal data from the cohorts. The method used to develop the EHII is described in detail in the paper by Pizzi et al “Measuring Child Socio-Economic Position in Birth Cohort Research: The Development of a Novel Standardized Household Income Indicator” (Int J Environ Res Public Health, 2020).

In brief, the cohort-specific EHII is derived according to the following steps: (i) identification of the household income potential predictors available both in the country-specific 2011 EUSILC database and in the cohort; (ii) construction of the prediction model and estimation of the coefficients; (iii) model validation (external validation) using the country-specific 2015 EUSILC database; (iv) whenever the model performs sufficiently well, derivation of the EHII indicator applying the regression coefficients obtained from the prediction model to the cohort data.

Since the equivalised total disposable household income (outcome) has a severely positively skewed distribution, multivariable linear regression models with log-transformation of the outcome are used to predict the income.

The EHII at time of birth/pregnancy is available for several birth cohorts member of the EU Child Cohort Network

| ***Table 1:* Subscales and factors of HEFAS 7-11** |  |
| --- | --- |
| **Subscale 1: Promotion of Cognitive and Linguistic Development** | **(PCLD)** |
| 1.1. Presence of Learning Materials | (PLM) |
| 1.2. Cognitive and Linguistic Scaffolding | (CLS) |
| 1.3. Encouraging Reading | (ER) |
| **Subscale 2: Promotion of Social and Emotional Development** | **(PSED)** |
| 2.1. Emotional Expressiveness | (EE) |
| 2.2. Setting of Limits and Optimal Frustration | (SLOF) |
| 2.3. Fostering Autonomy and Self-esteem | (FAS) |
| 2.4. Precedents of Self-Regulated Learning | (PSRL) |
| 2.5. Quality of Sibling Relations | (QSR) |
| **Subscale 3: Organisation of the Physical Environment and Social Context** | **(OPESC)** |
| 3.1. Quality of the Physical Environment | (QPE) |
| 3.2. Social Support Networks | (SSN) |
| 3.3. Promotion of Child's Social Relationships | (PCSR) |
| 3.4. Relations with the School | (RS) |
| **Subscale 4: Parental Stress and Conflict** | **(PSC)** |
| 4.1. Low Parental Stress | (LPS) |
| 4.2. Low Frequency of and Exposure to Conflict | (LFEC) |
| 3.3. Conflict Resolution | (CR) |
| **Subscale 5: Parental Profile Fostering Child Development** | **(PPFCD)** |
| 5.1. Parental Self-Efficacy | (Au) |
| 5.2. Knowledge regarding Development | (KD) |
| 5.3. Assertiveness | (As) |
| 5.4. Environmentalist Outlook on Development | (EOD) |
| 5.5. Involvement of the Father or Secondary Reference Figure | (FI) |

| **Supplementary Table 2: Minimal, adjusted and sensitivity models** | | | | | | | | | | | | | |
| --- | --- | --- | --- | --- | --- | --- | --- | --- | --- | --- | --- | --- | --- |
|  |  | Minimally adjusted models | | | | Fully adjusted models | | | | Sensitivity analyses | | | |
|  |  | B | lower | higher | p | B | lower | higher | p | B | lower | higher | p |
| Family social class | ref: Highest: I+II | 0 | 0 | 0 | 0 | 0 | 0 | 0 | 0 |  |  |  |  |
|  | Middle: III | -0.05 | -0.82 | 0.73 | 0.908 | -0.40 | -1.21 | 0.41 | 0.332 |  |  |  |  |
|  | Lowest: IV+V | -0.86 | -1.53 | -0.18 | 0.013 | -0.83 | -1.58 | -0.08 | 0.031 |  |  |  |  |
| Family education | ref: Both highly educated (he) | 0.00 | 0.00 | 0.00 | 0 | 0.00 | 0.00 | 0.00 | 0 |  |  |  |  |
|  | One he | -1.08 | -1.94 | -0.22 | 0.014 | -1.27 | -2.20 | -0.34 | 0.008 |  |  |  |  |
|  | None he | -0.96 | -1.79 | -0.13 | 0.023 | -0.87 | -1.80 | 0.05 | 0.063 |  |  |  |  |
| Family employment pregnancy | ref: Both employed | 0.00 | 0.00 | 0.00 | 0 | 0.00 | 0.00 | 0.00 | 0 |  |  |  |  |
|  | Not both employed | -0.75 | -1.50 | -0.01 | 0.048 | -0.59 | -1.40 | 0.21 | 0.149 |  |  |  |  |
| EHII pregnancy categorized | ref: Highest | 0.00 | 0.00 | 0.00 | 0 | 0.00 | 0.00 | 0.00 | 0 |  |  |  |  |
|  | Middle | -0.35 | -0.98 | 0.29 | 0.285 | -0.27 | -0.96 | 0.42 | 0.443 |  |  |  |  |
|  | Lowest | -1.00 | -2.14 | 0.14 | 0.085 | -0.89 | -2.13 | 0.36 | 0.161 |  |  |  |  |
| AROPE | ref: No risk | 0.00 | 0.00 | 0.00 | 0 | 0.00 | 0.00 | 0.00 | 0 | 0.00 | 0.00 | 0.00 | 0 |
|  | Risk | -0.14 | -1.01 | 0.73 | 0.755 | 0.14 | -0.77 | 1.04 | 0.763 | 0.26 | -0.66 | 1.19 | 0.578 |
| Risk of poverty | ref: No risk | 0.00 | 0.00 | 0.00 | 0 | 0.00 | 0.00 | 0.00 | 0 | 0.00 | 0.00 | 0.00 | 0 |
|  | Risk | -0.17 | -1.12 | 0.78 | 0.726 | 0.18 | -0.80 | 1.17 | 0.716 | 0.34 | -0.66 | 1.34 | 0.503 |
| Work intensity | ref: No risk | 0.00 | 0.00 | 0.00 | 0 | 0.00 | 0.00 | 0.00 | 0 | 0.00 | 0.00 | 0.00 | 0 |
|  | Risk | -0.02 | -1.40 | 1.35 | 0.977 | 0.21 | -1.24 | 1.66 | 0.773 | 0.41 | -1.07 | 1.89 | 0.589 |
| Material deprivation | ref: No risk | 0.00 | 0.00 | 0.00 | 0 | 0.00 | 0.00 | 0.00 | 0 | 0.00 | 0.00 | 0.00 | 0 |
|  | Risk | -0.61 | -1.97 | 0.74 | 0.373 | -0.54 | -1.93 | 0.85 | 0.446 | -0.46 | -1.87 | 0.95 | 0.524 |
| Minimally adjusted models include sex, age and cohort. |  |  |  |  |  |  |  |  |  |  |  |  |  |
| Adjusted models also include: parity, parental origin, parental age, and maternal history of pre-pregnancy anxiety and depression and maternal intelligence. | | | | | | | | | | | | |  |
| Sensitivity models additionally adjusted for main care provider, current family structure, and number of siblings at age 4-5. | | | | | | | | |  |  |  |  |  |

| **Supplementary Table 3: SEP relation to HEFAS 7-11** | | | | | | | | | | | | | | | | | | | | | |
| --- | --- | --- | --- | --- | --- | --- | --- | --- | --- | --- | --- | --- | --- | --- | --- | --- | --- | --- | --- | --- | --- |
|  |  | Subscale 1 | | | | Subscale 2 | | | | Subscale 3 | | | | Subscale 4 | | | | Subscale 5 | | | |
|  |  | B | lower | higher | p | B | lower | higher | p | B | lower | higher | p | B | lower | higher | p | B | lower | higher | p |
| Family social class | ref: Highest: I+II | 0 | 0 | 0 | 0 | 0 | 0 | 0 | 0 | 0 | 0 | 0 | 0 | 0 | 0 | 0 | 0 | 0 | 0 | 0 | 0 |
|  | Middle: III | -2.91 | -5.37 | -0.45 | 0.020 | -0.55 | -2.19 | 1.09 | 0.511 | 0.26 | -1.19 | 1.70 | 0.726 | -1.48 | -3.54 | 0.59 | 0.160 | -3.02 | -4.85 | -1.19 | 0.001 |
|  | Lowest: IV+V | -4.79 | -7.07 | -2.51 | <0.001 | -2.19 | -3.72 | -0.67 | 0.005 | -1.57 | -2.91 | -0.23 | 0.021 | -2.90 | -4.81 | -0.99 | 0.003 | -4.67 | -6.38 | -2.96 | <0.001 |
| Family education | ref: Both highly educated (he) | 0.00 | 0.00 | 0.00 | 0 | 0.00 | 0.00 | 0.00 | 0 | 0.00 | 0.00 | 0.00 | 0 | 0.00 | 0.00 | 0.00 | 0 | 0.00 | 0.00 | 0.00 | 0 |
|  | One he | -2.62 | -5.46 | 0.23 | 0.071 | -1.29 | -3.19 | 0.61 | 0.182 | -0.29 | -1.96 | 1.37 | 0.730 | -1.98 | -4.34 | 0.38 | 0.100 | -2.51 | -4.64 | -0.39 | 0.021 |
|  | None he | -5.31 | -8.12 | -2.50 | <0.001 | -1.44 | -3.32 | 0.44 | 0.134 | -0.27 | -1.92 | 1.37 | 0.742 | -1.75 | -4.08 | 0.57 | 0.139 | -4.19 | -6.28 | -2.10 | <0.001 |
| Family employment pregnancy | ref: Both employed | 0.00 | 0.00 | 0.00 | 0 | 0.00 | 0.00 | 0.00 | 0 | 0.00 | 0.00 | 0.00 | 0 | 0.00 | 0.00 | 0.00 | 0 | 0.00 | 0.00 | 0.00 | 0 |
|  | Not both employed | -1.61 | -4.08 | 0.86 | 0.200 | -0.83 | -2.47 | 0.82 | 0.323 | -1.00 | -2.44 | 0.44 | 0.174 | -1.74 | -3.84 | 0.35 | 0.103 | -2.46 | -4.32 | -0.59 | 0.010 |
| EHII pregnancy categorized | ref: Highest | 0.00 | 0.00 | 0.00 | 0 | 0.00 | 0.00 | 0.00 | 0 | 0.00 | 0.00 | 0.00 | 0 | 0.00 | 0.00 | 0.00 | 0 | 0.00 | 0.00 | 0.00 | 0 |
|  | Middle | -4.19 | -6.29 | -2.10 | <0.001 | -1.30 | -2.71 | 0.10 | 0.069 | -0.20 | -1.43 | 1.04 | 0.756 | -1.45 | -3.19 | 0.28 | 0.100 | -3.90 | -5.47 | -2.33 | <0.001 |
|  | Lowest | -6.92 | -10.68 | -3.17 | <0.001 | -2.03 | -4.57 | 0.51 | 0.116 | -1.10 | -3.32 | 1.12 | 0.331 | -3.88 | -7.10 | -0.66 | 0.018 | -5.02 | -7.86 | -2.18 | 0.001 |
| AROPE | ref: No risk | 0.00 | 0.00 | 0.00 | 0 | 0.00 | 0.00 | 0.00 | 0 | 0.00 | 0.00 | 0.00 | 0 | 0.00 | 0.00 | 0.00 | 0 | 0.00 | 0.00 | 0.00 | 0 |
|  | Risk | -1.10 | -3.90 | 1.70 | 0.441 | 1.02 | -0.85 | 2.89 | 0.284 | -0.06 | -1.68 | 1.57 | 0.947 | -2.22 | -4.68 | 0.25 | 0.078 | -1.69 | -3.84 | 0.45 | 0.121 |
| Risk of poverty | ref: No risk | 0.00 | 0.00 | 0.00 | 0 | 0.00 | 0.00 | 0.00 | 0 | 0.00 | 0.00 | 0.00 | 0 | 0.00 | 0.00 | 0.00 | 0 | 0.00 | 0.00 | 0.00 | 0 |
|  | Risk | -2.42 | -5.46 | 0.63 | 0.120 | 1.26 | -0.77 | 3.30 | 0.223 | -0.21 | -1.99 | 1.56 | 0.813 | -2.87 | -5.60 | -0.14 | 0.039 | -2.61 | -4.96 | -0.26 | 0.030 |
| Work intensity | ref: No risk | 0.00 | 0.00 | 0.00 | 0 | 0.00 | 0.00 | 0.00 | 0 | 0.00 | 0.00 | 0.00 | 0 | 0.00 | 0.00 | 0.00 | 0 | 0.00 | 0.00 | 0.00 | 0 |
|  | Risk | -1.68 | -6.15 | 2.80 | 0.463 | -0.39 | -3.46 | 2.68 | 0.803 | -2.54 | -5.11 | 0.03 | 0.053 | -6.24 | -10.39 | -2.09 | 0.003 | -3.41 | -6.79 | -0.02 | 0.048 |
| Material deprivation | ref: No risk | 0.00 | 0.00 | 0.00 | 0 | 0.00 | 0.00 | 0.00 | 0 | 0.00 | 0.00 | 0.00 | 0 | 0.00 | 0.00 | 0.00 | 0 | 0.00 | 0.00 | 0.00 | 0 |
|  | Risk | -1.86 | -6.10 | 2.38 | 0.390 | -0.16 | -2.97 | 2.64 | 0.909 | -3.13 | -5.60 | -0.67 | 0.013 | -4.12 | -7.82 | -0.41 | 0.030 | -4.99 | -8.24 | -1.74 | 0.003 |
| Adjusted for sex, age, cohort, parity, parental origin, parental age, and maternal history of pre-pregnancy anxiety and depression and maternal intelligence. | | | | | | | | | | | | | | | | | | | | | |

| **Supplementary Table 4: HEFAS 7-11 subscales relation to cognitive development (Raven’s CPM)** | | | | |
| --- | --- | --- | --- | --- |
|  |  | 95% CI | |  |
| Subscale | B | lower | higher | *p-value* |
| Subscale 1. Cognitive | 0.03 | 0.00 | 0.05 | 0.218 |
| Subscale 2. Emotional | 0.03 | 0.00 | 0.07 | 0.158 |
| Subscale 3. Organisation | -0.01 | -0.06 | 0.03 | 0.198 |
| Subscale 4. Stress | 0.03 | -0.01 | 0.06 | 0.166 |
| Subscale 5. Parenting | 0.05 | 0.02 | 0.09 | 0.138 |
| Adjusted for sex, age, cohort, parity, parental origin, parental age, and maternal history of pre-pregnancy anxiety and depression and maternal intelligence | | | | |
